# Supplementary material for: Coevolving residues distant from the ligand binding site are involved in GAF domain function
Source: Commun Chem. 2025 Apr 7;8:107. doi: 10.1038/s42004-025-01447-9 (PMC11977230; doi:10.1038/s42004-025-01447-9)
Supplement: Supplementary file 2 — Supplemental material [file 42004_2025_1447_MOESM2_ESM.pdf]

Supplementary Information

Coevolving Residues Distant from the Ligand Binding Site are Involved in GAF Domain Function

Wesam S. Ahmed[1], Anupriya M Geethakumari[1], Asfia Sultana[1], Anmol Tiwari[2], Tausif Altamash[1], Najla Arshad[3], Sandhya S Visweswariah[2], Kabir H Biswas[1,*]

Affiliations:
[1]Division of Biological and Biomedical Sciences, College of Health & Life Sciences, Hamad Bin Khalifa University, Qatar Foundation, Doha – 34110, Qatar
[2]Department of Developmental Biology and Genetics, Indian Institute of Science, Bengaluru – 560012, India
[3]Department of Immunobiology, Yale University School of Medicine, New Haven, CT 06520
[#]Current affiliation: Materials Science and Nano-Engineering (MSN) Department, Mohammed VI Polytechnic University (UM6P), Lot 660-Hay Moulay Rachid, Ben Guerir 43150, Morocco

***Correspondence:** kbiswas@hbku.edu.qa

ORCID:
Wesam S. Ahmed: 0000-0002-3441-2631
Anupriya M Geethakumari: 0000-0002-8932-9114
Asfia Sultan: 0000-0001-5454-1097
Tausif Altamash: 0000-0001-8856-0894
Najla Arshad: 0000-0001-5553-311X
Sandhya S. Visweswariah: 0000-0003-2786-7344
Kabir H Biswas: 0000-0001-9194-4127

Supplementary Methods

Bioinformatics analysis

We performed multiple sequence alignment of GAF domain-containing human PDEs (PDE2, 5, 6, 10, and 11) to see if the two residues are conserved in other human GAF domains. Additionally, we utilized the Genome Aggregation Database (gnomAD) [1] to identify missense, insertion, or deletion variants that carry mutations at the selected coevolving residue positions. Pathogenesis potential of these variants was assessed using in silico prediction tools, including CAAD [2], REVEL [3], and PolyPhen [4]. We applied a cutoff of 20 [2, 5], 0.5 [6], and 0.908 [6] for the CAAD, REVEL, and PolyPhen prediction scores, respectively, to distinguish potentially harmful variants from benign ones.

The effect of mutating the coevolving residue positions on the protein stability was investigated by calculating Gibbs free energy changes ($\Delta\Delta G$) using in silico structure- and sequence-based prediction tools including DynaMut [7], mCSM [8], SDM [9], DUET [10], DeepDDG [11], iDeepDDG [11], Maestro [12], PoPMuSiC [13], I-Mutant 2.0 [14], CUPSAT [15], Mupro [16], and ENCoM [17]. To probe the effect of the mutations on the protein flexibility, the elastic network contact model (ENCoM)-based vibrational entropy difference (ΔΔSvib) between WT and mutants was calculated [17]. Solvent accessibility of the mutated residues was predicted using PoPMuSiC online tool [13]. Arpeggio webserver [18] was used to calculate and compare the number of intramolecular interactions formed by the coevolving residue positions. Moreover, the impact of the mutants on the domain function was predicted using the SNAP2 tool [19]. SNAP2 prediction scores reflect the likelihood of the specific mutation to alter the native protein function and range from -100 (strong neutral prediction) to +100 (strong effect prediction). The scores can be interpreted as strong signal of effect (>+50), weak signal (-50<score<+50), or strong signal of neutral or no effect (<-50). We also predicted the impact of the L267A and F295A mutation using the ESM variant analysis [20], which revealed a moderately disruptive effect for the L267A mutation (log-likelihood ratio, LRR, value of -16.198) and a highly disruptive effect of the F295A mutation (LRR value of -19.657) on the PDE5 GAFa domain function.

Supplementary Results

Bioinformatics analysis

Because our coevolutionary analysis suggested that the two coevolving residue positions may play a role in the GAF domain function, we wanted to see if these two residues are conserved in other human GAF domains. To achieve this, we performed multiple sequence alignment of GAF domain-containing human PDEs (PDE2, 5, 6, 10, and 11). The alignment showed that these two residues are indeed conserved across both the GAFa and GAFb domains of the cyclic nucleotide-binding human PDEs (Supplementary Text 1), suggesting an essential role that these two residues may play in the function of the associated PDEs. To further confirm their importance, we assessed if there are any reported variants that involve mutations at these two locations in the PDE5 GAF domains. To achieve this, we searched for missense, insertion, or deletion variants in the gnomAD database [1]. We listed these variants in Supplementary Table 2. Assessment of the deleterious effect of these variants using the in silico prediction tools CADD [2], REVEL [3], and PolyPhen [4] suggested that all variants have deleterious effects on the associated PDE5A gene, confirming the potentially essential role of these two residue positions on the associated PDE5A gene (Supplementary Table 2).

Moreover, we utilized in silico prediction tools to explore the impact of mutating the two positions on the PDE5 GAFa domain stability, flexibility, and functionality. Mutating either of the two positions was predicted by the SNAP2 tool to have a functionally deleterious effect. More specifically, our analysis showed that mutating either of the two residues results in a destabilizing effect on the domain. Additionally, there was an increase in the overall flexibility of the domain upon mutating the residue positions, with more flexibility observed for the F295A mutant (Supplementary Table 4). This increase in flexibility was most observed for the α4 and terminal helices (Supplementary Figure 9A). To explore how these effects are brought into display, we compared the number of contact interactions formed by these two positions in the WT and mutant domains. We found that mutating these two positions drastically affects the number of hydrophobic contacts they form with neighboring residues (Supplementary Table 5; Supplementary Figure 9B). Moreover, both residues are hydrophobic and, therefore, can be affected negatively by solvent accessibility. The PoPMuSiC tool predicted the 295 positions to have more solvent accessibility, compared to 0.0% accessibility for the 267 positions, which may contribute to the more profound effect observed upon mutating this position on the domain's structure and function. Overall, our in silico analysis shows that mutating the two coevolving residue positions is predicted to have drastic effects on the stability, flexibility, and functionality of the GAF domain facilitated by the excessive loss of hydrophobic contacts formed by the two residue positions.

Supplementary Texts

Supplementary Text 1: Multiple sequence alignment of GAF domain-containing human PDEs showing that the L267 and F295 positions are conserved in both GAFa and GAFb domains of all GAF domain-containing human PDEs.

```
PDE6C         -------------------------------------------MGE-----INQVAVEKY    12
PDE6A         -------------------------------------------MGE-----VTAEEVEKF    12
PDE6B         --------------------------------------------MS-----LSEEQARSF    11
PDE5A1        -----------------MERAGPSFGQQ-----RQQQQPQQQKQQQ-----RDQDSVEAW    33
PDE11A1       -----------------------------------------MAASR-----LDFGEVETF    14
PDE10A1       ------------------------------------------------------------    0
PDE2A1        MGQACGHSILCRSQQYPAARPAEPRGQQVFLKPDEPPPPPQPCADSLQDALLSLGSVIDI    60


PDE6C         LEENPQFAKEYFDRKLRVEVL---------------------------------------    33
PDE6A         LDSNIGFAKQYYNLHYRAKLI---------------------------------------    33
PDE6B         LDQNPDFARQYFGKKLSPENV---------------------------------------    32
PDE5A1        LDDHWDFTFSYFVRKATREMVNAWFAERVHTIP-----------------VCKEGIR--G    74
PDE11A1       LDRHPELFEDYLMRKGKQEMVEKWLQRHSQGQGALGPRPSLAGTSSLAHSTCRGGSSVGG    74
PDE10A1       -------------------------------------------------------MRIEE    5
PDE2A1        SGLQRAVKEALSAVLPRVETVYTYLLDGESQLVCEDPPHELPQE-----GKVREAIISQK    115


PDE6C         ------------------------------------------------------------    33
PDE6A         ------------------------------------------------------------    33
PDE6B         ------------------------------------------------------------    32
PDE5A1        HT---------------ESCSCPLQQSPRADNSAPGTPTRKISA------SEFDRPLR--    111
PDE11A1       GTGPNGSAHSQPLPGGGDCGGVPLSPSWAGGSRGDGNLQRRASQ------KELRKSFARS    128
PDE10A1       RKSQHL----------------------TGLTDE------KVKAYLSLHPQVLDE-----    32
PDE2A1        RLGCNG----------------------LGFSDLPGKPLARLVAPLAPDTQVLVMPLADK    153


PDE6C         --------------------------------------------GEIF---K----NSQV    42
PDE6A         --------------------------------------------SDLL---G----AKEA    42
PDE6B         --------------------------------------------AAAC--------EDGC    40
PDE5A1        ----------PIVVKDSEGT--------------------VSFLS-DS---EKKEQMPLT    137
PDE11A1       KAIHVNRTYDEQVTSRAQEPLSSVRRRALLRKASSLPPTTAHILSALL---ESRVNLPRY    185
PDE10A1       -----------FVSE--SVSAETVEK-WLKRKN------NK----------SEDESAPKE    62
PDE2A1        EAGAVAAV---ILVH--CGQLSDNEE-WSLQAV------EKHTLVALRRVQVLQQRGPRE    201


PDE6C         PVQSSMS--------------F--SELTQVEESALCLELLWTV---QEEGGTPEQGVHRA    83
PDE6A         AVDF-SN--------------Y--HSPSSMEESEIIFDLLRDF---QENL-QTEKCIFNV    81
PDE6B         PPDC-DS--------------L--RDLCQVEESTALLELVQDM---QESI-NMERVVFKV    79
PDE5A1        PPRF---------------------DHDEGDQCSRLLELVKDI---SSHL-DVTALCHKI    172
PDE11A1       PPTAIDY--------------K--CHLKKHNERQFFLELVKDI---SNDL-DLTSLSYKI    225
PDE10A1       VSR------------------YQDT-----NMQGVVYELNSYIEQRLDTGGDNQLLLYEL    99
PDE2A1        APRAVQNPPEGTAEDQKGGAAYTDRDRKILQLCGELYDLDASS------------LQLKV    249
                                            :      :*                   .

PDE6C         LQRLAHLLQADRCSMFLCR-SRNGIPEVASRLLDVTPTSKFEDNLVG-PDKEVVFPL---    138
PDE6A         MKKLCFLLQADRMSLFMYR-TRNGIAELATRLFNVHKDAVLEDCLVM-PDQEIVFPL---    136
PDE6B         LRRLCTLLQADRCSLFMYR-QRNGVAELATRLFSVQPDSVLEDCLVP-PDSEIVFPL---    134
PDE5A1        FLHIHGLISADRYSLFLVCEDSSNDKFLISRLFDVAEGSTLEEV----SNNCIRLEW---    225
PDE11A1       LIFVCLMVDADRCSLFLVEGAAAGKKTLVSKFFDVHAGTPLLPCSSTENSNEVQVPW---    282
PDE10A1       SSIIKIATKADGFALYFLGECNNSL-CIFT------PPGI-------KEGKPRLIPAGPI    145
PDE2A1        LQYLQQETRASRCCLLLVSEDNLQLSCKVI------GDKV--------LGEEVSFPL---    292
                 :     *.   .: :                                ..   .
```

GAFa – L267 (PDE5A1 numbering) ↓

```
PDE6C         --DIGIVGWAAHTKKTHNVPDVKKNSHFSDFMDKQTGYVTKNLLATPIVVGK-EVLAVI-    194
PDE6A         --DMGIVGHVAHSKKIANVPNTEEDEHFCDFVDILTEYKTKNILASPIMNGK-DVVAII-    192
PDE6B         --DIGVVGHVAQTKKMVNVEDVAECPHFSSFADELTDYKTKNMLATPIMNGK-DVVAVI-    190
PDE5A1        --NKGIVGHVAALGEPLNIKDAYEDPRFNAEVDQITGYKTQSILCMPIKNHREEVVGVA-    282
```

```
PDE11A1       --GKGIIGYVGEHGETVNIPDAYQDRRFNDEIDKLTGYKTKSLLCMPIRSSDGEIIGVA-   339
PDE10A1       TQGTTVSAYVAKSRKTLLVEDILGDERFPRGTGLESGTRIQSVLCLPIVTAIGDLIGIL-   204
PDE2A1        T---GCLGQVVEDKKSIQLKDLTSEDV--QQLQSMLGCELQAMLCVPVISRATDQVVALA   347
                     . .    :  :  :                   : :*. *:     : :
```

↓ **GAFa - F295 (PDE5A1 numbering)**

```
PDE6C         MAVNKV--NASEFSKQDEEVFSKYLNFVSIILRLHHTSYMYNIESRRSQILMWSANKVFE   252
PDE6A         MAVNKV--DGSHFTKRDEEILLKYLNFANLIMKVYHLSYLHNCETRRGQILLWSGSKVFE   250
PDE6B         MAVNKL--NGPFFTSEDEDVFLKYLNFATLYLKIYHLSYLHNCETRRGQVLLWSANKVFE   248
PDE5A1        QAINKKSGNGGTFTEKDEKDFAAYLAFCGIVLHNAQLYETSLLENKRNQVLLDLASLIFE   342
PDE11A1       QAINKI-PEGAPFTEDDEKVMQMYLPFCGIAISNAQLFAASRKEYERSRALLEVVNDLFE   398
PDE10A1       ELYRHW--GKEAFCLSHQEVATANLAWASVAIHQVQVCRGLAKQTELNDFLLDVSKTYFD   262
PDE2A1        CAFNKL--EGDLFTDEDEHVIQHCFHYTSTVLTSTLAFQKEQKLKCECQALLQVAKNLFT   405
                 .:       *   .:.     : :    :                  *:   .  *

PDE6C         ELTDVERQFHKALYTVRSYLNCERYSIGLLDMTKEKE--FYDEWPIKLGEVEPYKGPKTP   310
PDE6A         ELTDIERQFHKALYTVRAFLNCDRYSVGLLDMTKQKE--FFDVWPVLMGEVPPYSGPRTP   308
PDE6B         ELTDIERQFHKAFYTVRAYLNCERYSVGLLDMTKEKE--FFDVWSVLMGESQPYSGPRTP   306
PDE5A1        EQQSLEVILKKIAATIISFMQVQKCTIFIVDEDCSDS--FSSVFHM-----------ECE   389
PDE11A1       EQTDLEKIVKKIMHRAQTLLKCERCSVLLLEDIESPVVKFTKSFELM--------SPKCS   450
PDE10A1       NIVAIDSLLEHIMIYAKNLVNADRCALFQVDHKNKEL--YSDLFDIG----------EEK   310
PDE2A1        HLDDVSVLLQEIITEARNLSNAEICSVFLLDQN--EL--VAKVFDG--------------   447
              .   :.  ...         : :  ::  ::          . :

PDE6C         DGREVNFYKIIDYILHGKEEIKVIPTPPADHWTLISGLPTYVAENGFICNMMNAPADEYF   370
PDE6A         DGREINFYKVIDYILHGKEDIKVIPNPPPDHWALVSGLPAYVAQNGLICNIMNAPAEDFF   368
PDE6B         DGREIVFYKVIDYVLHGKEEIKVIPTPSADHWALASGLPSYVAESGFICNIMNASADEMF   366
PDE5A1        ELEKS-------------SD--TLT-REHDANKINYMYAQYVKNTMEPLNIPDVSKDKRF   433
PDE11A1       ADAEN-------------SFKESMEKSSYSDWLINNSIAELVASTGLPVNISDAYQDPRF   497
PDE10A1       ------------------EGKPVFKKTKEIRFSIEKGIAGQVARTGEVLNIPDAYADPRF   352
PDE2A1        --------------------GVVDDESYEIRIPADQGIAGHVATTGQILNIPDAYAHPLF   487
                                                       *  .    *: :.  .  *
```

GAFb - L452 (PDE5A1 numbering) ↓ **GAFb – F484 (PDE5A1 numbering)** ↓

```
PDE6C         TFQKGPV-DETGWVIKNVLSLPIVNKK-EDIVGVATFYNRKD-----GKPFDEHDEYITE   423
PDE6A         AFQKEPL-DESGWMIKNVLSMPIVNKK-EEIVGVATFYNRKD-----GKPFDEMDETLME   421
PDE6B         KFQEGAL-DDSGWLIKNVLSMPIVNKK-EEIVGVATFYNRKD-----GKPFDEQDEVLME   419
PDE5A1        PWTTENTGNVNQQCIRSLLCTPIKNGKKNKVIGVCQLVNKMEENTGKVKPFNRNDEQFLE   493
PDE11A1       DAEA---DQISGFHIRSVLCVPIWNSN-HQIIGVAQVLNRLD-----GKPFDDADQRLFE   548
PDE10A1       NREV---DLYTGYTTRNILCMPIVSR--GSVIGVVQMVNKIS-----GSAFSKTDENNFK   402
PDE2A1        YRGV---DDSTGFRTRNILCFPIKNEN-QEVIGVAELVNKIN-----GPWFSKFDEDLAT   538
                        .    :.:*. ** .    .::**  . *: .        *.  *:

PDE6C         TLTQFLGWSLLNTDTYDKMNKLENRKDIAQEMLMNQTKATPEEIKSILKFQEKLNVDVID   483
PDE6A         SLTQFLGWSVLNPDTYESMNKLENRKDIFQDIVKYHVKCDNEEIQKILKTREVYGKE-PW   480
PDE6B         SLTQFLGWSVMNTDTYDKMNKLENRKDIAQDMVLYHVKCDRDEIQLILPTRARLGKE-PA   478
PDE5A1        AFVIFCGLGIQNTQMYEAVERAMAKQMVTLEVLSYHASAAEEETRELQS-----------   542
PDE11A1       AFVIFCGLGINNTIMYDQVKKSWAKQSVALDVLSYHATCSKAEVD---K-----------   594
PDE10A1       MFAVFCALALHCANMYHRIRHSECIYRVTMEKLSYHSICTSEEWQGLMQ-----------   451
PDE2A1        AFSIYCGISIAHSLLYKKVNEAQYRSHLANEMMMYHMKVSDDEYTKLLH-----------   587
               :  : . .:     *. :..      :  : :  :      *

PDE6C         DCEEKQLVAILKEDLPDPR--SAELYEFRFSDFPLTEHGLIKCGIRLFFEINVVEKFKVP   541
PDE6A         ECEEEELAEILQAELPDAD--KYEINKFHFSDLPLTELELVKCGIQMYYELKVVDKFHIP   538
PDE6B         DCDEDELGEILKEELPGPT--TFDIYEFHFSDLECTELDLVKCGIQMYYELGVVRKFQIP   536
PDE5A1        ---------LAAAVVPSAQ--TLKITDFSFSDFELSDLETALCTIRMFTDLNLVQNFQMK   591
PDE11A1       ---------FKAANIPLVS--ELAIDDIHFDDFSLDVDAMITAALRMFMELGMVQKFKID   643
PDE10A1       ------------FTLPVRLCKEIELFHFDIGPFE----NMWPGIFVYMV-HRSCGTSCFE   494
PDE2A1        ------------DGIQPVAAIDSNFASFTYTPRSLPEDDTSMAILSMLQDMNFINNYKID   635
                            :         :  :                :          .  .

PDE6C         VEVLTRWMYTVRKGYRA-VTYHNWRHGFNVGQTMFTLLMTGRLKKYYTDLEAFAMLAAAF   600
PDE6A         QEALVRFMYSLSKGYRK-ITYHNWRHGFNVGQTMFSLLVTGKLKRYFTDLEALAMVTAAF   597
PDE6B         QEVLVRFLFSISKGYRR-ITYHNWRHGFNVAQTMFTLLMTGKLKSYYTDLEAFAMVTAGL   595
PDE5A1        HEVLCRWILSVKKNYRKNVAYHNWRHAFNTAQCMFAALKAGKIQNKLTDLEILALLIAAL   651
PDE11A1       YETLCRWLLTVRKNYRM-VLYHNWRHAFNVCQLMFAMLTTAGFQDILTEVEILAVIVGCL   702
PDE10A1       LEKLCRFIMSVKKNYRR-VPYHNWKHAVTVAHCMYAILQNN--HTLFTDLERKGLLIACL   551
PDE2A1        CPTLARFCLMVKKGYRD-PPYHNWMHAFSVSHFCYLLYKNLELTNYLEDIEIFALFISCM   694
                 * *:   : *.**    **** *.... :  :             ::*  .:. . :
```

```
PDE6C        CHDIDHRGTNNLYQMKSTSPLARLHGSS--ILERHHLEYSKTLLQDESLNIFQNLNKRQF    658
PDE6A        CHDIDHRGTNNLYQMKSQNPLAKLHGSS--ILERHHLEFGKTLLRDESLNIFQNLNRRQH    655
PDE6B        CHDIDHRGTNNLYQMKSQNPLAKLHGSS--ILERHHLEFGKFLLSEETLNIYQNLNRRQH    653
PDE5A1       SHDLDHRGVNNSYIQRSEHPLAQLYCHS--IMEHHHFDQCLMILNSPGNQILSGLSIEEY    709
PDE11A1      CHDLDHRGTNNAFQAKSGSALAQLYGTS-ATLEHHHFNHAVMILQSEGHNIFANLSSKEY    761
PDE10A1      CHDLDHRGFSNSYLQKFDHPLAALYST--STMEQHHFSQTVSILQLEGHNIFSTLSSSEY    609
PDE2A1       CHDLDHRGTNNSFQVASKSVLAALYSSEGSVMERHHFAQAIAILNTHGCNIFDHFSRKDY    754
             .**:**** .* :       ** *:      :*:**:     :*     :*   :.  :.

PDE6C        ETVIHLFEVAIIATDLALYFKKRTMFQKIVDACEQMQTEEEAIKYVTVDPTKKEIIMAMM    718
PDE6A        EHAIHMMDIAIIATDLALYFKKRTMFQKIVDQSKTYESEQEWTQYMMLEQTRKEIVMAMM    715
PDE6B        EHVIHLMDIAIIATDLALYFKKRAMFQKIVDESKNYQDKKSWVEYLSLETTRKEIVMAMM    713
PDE5A1       KTTLKIIKQAILATDLALYIKRRGEFFELIRKN----------QFNLEDPHQKELFLAML    759
PDE11A1      SDLMQLLKQSILATDLTLYFERRTEFFELVSKG----------EYDWNIKNHRDIFRSML    811
PDE10A1      EQVLEIIRKAIIATDLALYFGNRKQLEEMYQTG----------SLNLNNQSHRDRVIGLM    659
PDE2A1       QRMLDLMRDIILATDLAHHLRIFKDLQKMAEVG----------YD-RNNKQHHRLLLCLL    803
             .  :.::   *:****: ::     : ::                      ::  .  ::

PDE6C        MTACDLSAITKPWEVQSQVALMVANEFWEQGDLERTVLQQQPIPMMDRNKRDELPKLQVG    778
PDE6A        MTACDLSAITKPWEVQSQVALLVAAEFWEQGDLERTVLQQNPIPMMDRNKADELPKLQVG    775
PDE6B        MTACDLSAITKPWEVQSKVALLVAAEFWEQGDLERTVLDQQPIPMMDRNKAAELPKLQVG    773
PDE5A1       MTACDLSAITKPWPIQQRIAELVATEFFDQGDRERKELNIEPTDLMNREKKNKIPSMQVG    819
PDE11A1      MTACDLGAVTKPWEISRQVAELVTSEFFEQGDRERLELKLTPSAIFDRNRKDELPRLQLE    871
PDE10A1      MTACDLCSVTKLWPVTKLTANDIYAEFWAEGDEMK-KLGIQPIPMMDRDKKDEVPQGQLG    718
PDE2A1       MTSCDLSDQTKGWKTTRKIAELIYKEFFSQGDLEK-AMGNRPMEMMDREK-AYIPELQIS    861
             **:***   ** *      *  :  **: :**  :  :   *  :::*::   :*  *:

PDE6C        FIDFVCTFVYKEFSRFHKEITPMLSGLQNNRVEWKSLADEYDAKMKVIEEEAKKQEGGAE    838
PDE6A        FIDFVCTFVYKEFSRFHEEITPMLDGITNNRKEWKALADEYDAKMKVQEEKKQKQQSAKS    835
PDE6B        FIDFVCTFVYKEFSRFHEEILPMFDRLQNNRKEWKALADEYEAKVKALEEKEEEERVAAK    833
PDE5A1       FIDAICLQLYEALTHVSEDCFPLLDGCRKNRQKWQALAEQQEKMLINGESG-------Q-    871
PDE11A1      WIDSICMPLYQALVKVNVKLKPMLDSVATNRSKWEELHQKRLLASTASSSPASVM-VAK-    929
PDE10A1      FYNAVAIPCYTTLTQILPPTEPLLKACRDNLSQWEKVIRGEETATWISSPSVAQ--KAAA    776
PDE2A1       FMEHIAMPIYKLLQDLFPKAAELYERVASNREHWTKVSHKFTIRGLPSNNSLDF--LDEE    919
             : : :.   *  :  .      : .    *  .*  :           .

PDE6C        KAAEDSGG-----GDDKKSKTCLML---       858
PDE6A        AAAGNQPGGNPSPGGATTSKSCCIQ---       860
PDE6B        KVGTEICNGGPA----PKSSTCCIL---       854
PDE5A1       -AKRN-----------------------       875
PDE11A1      -EDRN-----------------------       933
PDE10A1      S--ED-----------------------       779
PDE2A1       YEVPDLD------GTRAPINGCCSLDAE       941
                 :
```

Supplementary Text 2: Tcl script used to calculate the free binding energy using the CaFE plugin in VMD.

```
package require cafe 1.0
mmpbsa -top complex.psf \
-trj complex.dcd \
-com "protein or resname cgmp" \
-rec "protein" \
-lig "resname cgmp" \
-out mmpbsa_1000ns.log \
-stride 1 \
-par par_all36_prot.prm -par toppar_water_ions_namd.str -par
toppar_all36_carb_glycopeptide.str -par par_all36_na.prm -par
par_all36_lipid.prm -par par_all36_lipid.prm -par par_all36_cgenff.prm -par
par_all36_carb.prm \
-par cgmp.prm \
-mm 1 \
-mm_exe ../namd2 \
-pb 2 \
-pb_exe ../apbs.exe \
-sa 1
quit
```

Supplementary Text 3: Protein sequence of GFP^2-GAFa-RLuc constructs.

GFP^2-GAFa-RLuc

MVSKGEELFTGVVPILVELDGDVNGHKFSVSGEGEGDATYGKLTLKFICTTGKLPVPWPTLVTTLSYGVQCFSRYPDHMKQHDFFKSAMPEGYVQER
TIFFKDDGNYKTRAEVKFEGDTLVNRIELKGIDFKEDGNILGHKLEYNYNSHNVYIMADKQKNGIKVNFKIRHNIEDGSVQLADHYQQNTPIGDGPV
LLPDNHYLSTQSALSKDPNEKRDHMVLLEFVTAAGITLGMDELYKSGSSLISSHLDVTALCHKIFLHIHGLISADRYSLFLVCEDSSNDKFLISRLF
DVAEGSTLEEVSNNCIRLEWNKGIVGHVAALGEPLNIKDAYEDPRFNAEVDQITGYKTQSILCMPIKNHREEVVGVAQAINKKSGNGGTFTEKDEKD
FAAYLAFCGIVLHNRSDIGPSRATMTSKVYDPEQRKRMITGPQWWARCKQMNVLDSFINYYDSEKHAENAVIFLHGNAASSYLWRHVVPHIEPVARC
IIPDLIGMGKSGKSGNGSYRLLDHYKYLTAWFELLNLPKKIIFVGHDWGACLAFHYSYEHQDKIKAIVHAESVVDVIESWDEWPDIEEDIALIKSEE
GEKMVLENNFFVETMLPSKIMRKLEPEEFAAYLEPFKEKGEVRRPTLSWPREIPLVKGGKPDVVQIVRNYNAYLRASDDLPKMFIESDPGFFSNAIV
EGAKKFPNTEFVKVKGLHFSQEDAPDEMGKYIKSFVERVLKNEQ*

GFP^2-GAFa(L267A)-RLuc

MVSKGEELFTGVVPILVELDGDVNGHKFSVSGEGEGDATYGKLTLKFICTTGKLPVPWPTLVTTLSYGVQCFSRYPDHMKQHDFFKSAMPEGYVQER
TIFFKDDGNYKTRAEVKFEGDTLVNRIELKGIDFKEDGNILGHKLEYNYNSHNVYIMADKQKNGIKVNFKIRHNIEDGSVQLADHYQQNTPIGDGPV
LLPDNHYLSTQSALSKDPNEKRDHMVLLEFVTAAGITLGMDELYKSGSSLISSHLDVTALCHKIFLHIHGLISADRYSLFLVCEDSSNDKFLISRLF
DVAEGSTLEEVSNNCIRLEWNKGIVGHVAALGEPLNIKDAYEDPRFNAEVDQITGYKTQSIACMPIKNHREEVVGVAQAINKKSGNGGTFTEKDEKD
FAAYLAFCGIVLHNRSDIGPSRATMTSKVYDPEQRKRMITGPQWWARCKQMNVLDSFINYYDSEKHAENAVIFLHGNAASSYLWRHVVPHIEPVARC
IIPDLIGMGKSGKSGNGSYRLLDHYKYLTAWFELLNLPKKIIFVGHDWGACLAFHYSYEHQDKIKAIVHAESVVDVIESWDEWPDIEEDIALIKSEE
GEKMVLENNFFVETMLPSKIMRKLEPEEFAAYLEPFKEKGEVRRPTLSWPREIPLVKGGKPDVVQIVRNYNAYLRASDDLPKMFIESDPGFFSNAIV
EGAKKFPNTEFVKVKGLHFSQEDAPDEMGKYIKSFVERVLKNEQ*

GFP^2-GAFa(F295A)-RLuc

MVSKGEELFTGVVPILVELDGDVNGHKFSVSGEGEGDATYGKLTLKFICTTGKLPVPWPTLVTTLSYGVQCFSRYPDHMKQHDFFKSAMPEGYVQER
TIFFKDDGNYKTRAEVKFEGDTLVNRIELKGIDFKEDGNILGHKLEYNYNSHNVYIMADKQKNGIKVNFKIRHNIEDGSVQLADHYQQNTPIGDGPV
LLPDNHYLSTQSALSKDPNEKRDHMVLLEFVTAAGITLGMDELYKSGSSLISSHLDVTALCHKIFLHIHGLISADRYSLFLVCEDSSNDKFLISRLF
DVAEGSTLEEVSNNCIRLEWNKGIVGHVAALGEPLNIKDAYEDPRFNAEVDQITGYKTQSILCMPIKNHREEVVGVAQAINKKSGNGGTATEKDEKD
FAAYLAFCGIVLHNRSDIGPSRATMTSKVYDPEQRKRMITGPQWWARCKQMNVLDSFINYYDSEKHAENAVIFLHGNAASSYLWRHVVPHIEPVARC
IIPDLIGMGKSGKSGNGSYRLLDHYKYLTAWFELLNLPKKIIFVGHDWGACLAFHYSYEHQDKIKAIVHAESVVDVIESWDEWPDIEEDIALIKSEE
GEKMVLENNFFVETMLPSKIMRKLEPEEFAAYLEPFKEKGEVRRPTLSWPREIPLVKGGKPDVVQIVRNYNAYLRASDDLPKMFIESDPGFFSNAIV
EGAKKFPNTEFVKVKGLHFSQEDAPDEMGKYIKSFVERVLKNEQ*

Supplementary Text 4: Protein sequence of GFP2-PDE5A2-RLuc constructs.

GFP²-PDE5A2-RLuc

MVSKGEELFTGVVPILVELDGDVNGHKFSVSGEGEGDATYGKLTLKFICTTGKLPVPWPTLVTTLSYGVQCFSRYPDHMKQHDFFKSAMPEGYVQER
TIFFKDDGNYKTRAEVKFEGDTLVNRIELKGIDFKEDGNILGHKLEYNYNSHNVYIMADKQKNGIKVNFKIRHNIEDGSVQLADHYQQNTPIGDGPV
LLPDNHYLSTQSALSKDPNEKRDHMVLLEFVTAAGITLGMDELYKSGLRSRRALELGSTSNGRQCAGIRLMLPFGDKTREMVNAWFAERVHTIPVCK
EGIRGHTESCSCPLQQSPRADNSAPGTPTRKISASEFDRPLRPIVVKDSEGTVSFLSDSEKKEQMPLTPPRFDHDEGDQCSRLLELVKDISSHLDVT
ALCHKIFLHIHGLISADRYSLFLVCEDSSNDKFLISRLFDVAEGSTLEEVSNNCIRLEWNKGIVGHVAALGEPLNIKDAYEDPRFNAEVDQITGYKT
QSILCMPIKNHREEVVGVAQAINKKSGNGGTFTEKDEKDFAAYLAFCGIVLHNAQLYETSLLENKRNQVLLDLASLIFEEQQSLEVILKKIAATIIS
FMQVQKCTIFIVDEDCSDSFSSVFHMECEELEKSSDTLTREHDANKINYMYAQYVKNTMEPLNIPDVSKDKRFPWTTENTGNVNQQCIRSLLCTPIK
NGKKNKVIGVCQLVNKMEENTGKVKPFNRNDEQFLEAFVIFCGLGIQNTQMYEAVERAMAKQMVTLEVLSYHASAAEEETRELQSLAAAVVPSAQTL
KITDFSFSDFELSDLETALCTIRMFTDLNLVQNFQMKHEVLCRWILSVKKNYRKNVAYHNWRHAFNTAQCMFAALKAGKIQNKLTDLEILALLIAAL
SHDLDHRGVNNSYIQRSEHPLAQLYCHSIMEHHHFDQCLMILNSPGNQILSGLSIEEYKTTLKIIKQAILATDLALYIKRRGEFFELIRKNQFNLED
PHQKELFLAMLMTACDLSAITKPWPIQQRIAELVATEFFDQGDRERKELNIEPTDLMNREKKNKIPSMQVGFIDAICLQLYEALTHVSEDCFPLLDG
CRKNRQKWQALAEQQEKMLINGESGQAKRYRGPGIPPARATMTSKVYDPEQRKRMITGPQWWARCKQMNVLDSFINYYDSEKHAENAVIFLHGNAAS
SYLWRHVVPHIEPVARCIIPDLIGMGKSGKSGNGSYRLLDHYKYLTAWFELLNLPKKIIFVGHDWGACLAFHYSYEHQDKIKAIVHAESVVDVIESW
DEWPDIEEDIALIKSEEGEKMVLENNFFVETMLPSKIMRKLEPEEFAAYLEPFKEKGEVRRPTLSWPREIPLVKGGKPDVVQIVRNYNAYLRASDDL
PKMFIESDPGFFSNAIVEGAKKFPNTEFVKVKGLHFSQEDAPDEMGKYIKSFVERVLKNEQ*

GFP²-PDE5A2(L267A)-RLuc

MVSKGEELFTGVVPILVELDGDVNGHKFSVSGEGEGDATYGKLTLKFICTTGKLPVPWPTLVTTLSYGVQCFSRYPDHMKQHDFFKSAMPEGYVQER
TIFFKDDGNYKTRAEVKFEGDTLVNRIELKGIDFKEDGNILGHKLEYNYNSHNVYIMADKQKNGIKVNFKIRHNIEDGSVQLADHYQQNTPIGDGPV
LLPDNHYLSTQSALSKDPNEKRDHMVLLEFVTAAGITLGMDELYKSGLRSRRALELGSTSNGRQCAGIRLMLPFGDKTREMVNAWFAERVHTIPVCK
EGIRGHTESCSCPLQQSPRADNSAPGTPTRKISASEFDRPLRPIVVKDSEGTVSFLSDSEKKEQMPLTPPRFDHDEGDQCSRLLELVKDISSHLDVT
ALCHKIFLHIHGLISADRYSLFLVCEDSSNDKFLISRLFDVAEGSTLEEVSNNCIRLEWNKGIVGHVAALGEPLNIKDAYEDPRFNAEVDQITGYKT
QSIACMPIKNHREEVVGVAQAINKKSGNGGTFTEKDEKDFAAYLAFCGIVLHNAQLYETSLLENKRNQVLLDLASLIFEEQQSLEVILKKIAATIIS
FMQVQKCTIFIVDEDCSDSFSSVFHMECEELEKSSDTLTREHDANKINYMYAQYVKNTMEPLNIPDVSKDKRFPWTTENTGNVNQQCIRSLLCTPIK
NGKKNKVIGVCQLVNKMEENTGKVKPFNRNDEQFLEAFVIFCGLGIQNTQMYEAVERAMAKQMVTLEVLSYHASAAEEETRELQSLAAAVVPSAQTL
KITDFSFSDFELSDLETALCTIRMFTDLNLVQNFQMKHEVLCRWILSVKKNYRKNVAYHNWRHAFNTAQCMFAALKAGKIQNKLTDLEILALLIAAL
SHDLDHRGVNNSYIQRSEHPLAQLYCHSIMEHHHFDQCLMILNSPGNQILSGLSIEEYKTTLKIIKQAILATDLALYIKRRGEFFELIRKNQFNLED
PHQKELFLAMLMTACDLSAITKPWPIQQRIAELVATEFFDQGDRERKELNIEPTDLMNREKKNKIPSMQVGFIDAICLQLYEALTHVSEDCFPLLDG
CRKNRQKWQALAEQQEKMLINGESGQAKRYRGPGIPPARATMTSKVYDPEQRKRMITGPQWWARCKQMNVLDSFINYYDSEKHAENAVIFLHGNAAS
SYLWRHVVPHIEPVARCIIPDLIGMGKSGKSGNGSYRLLDHYKYLTAWFELLNLPKKIIFVGHDWGACLAFHYSYEHQDKIKAIVHAESVVDVIESW
DEWPDIEEDIALIKSEEGEKMVLENNFFVETMLPSKIMRKLEPEEFAAYLEPFKEKGEVRRPTLSWPREIPLVKGGKPDVVQIVRNYNAYLRASDDL
PKMFIESDPGFFSNAIVEGAKKFPNTEFVKVKGLHFSQEDAPDEMGKYIKSFVERVLKNEQ*

GFP²-PDE5A2(F295A)-RLuc

MVSKGEELFTGVVPILVELDGDVNGHKFSVSGEGEGDATYGKLTLKFICTTGKLPVPWPTLVTTLSYGVQCFSRYPDHMKQHDFFKSAMPEGYVQER
TIFFKDDGNYKTRAEVKFEGDTLVNRIELKGIDFKEDGNILGHKLEYNYNSHNVYIMADKQKNGIKVNFKIRHNIEDGSVQLADHYQQNTPIGDGPV
LLPDNHYLSTQSALSKDPNEKRDHMVLLEFVTAAGITLGMDELYKSGLRSRRALELGSTSNGRQCAGIRLMLPFGDKTREMVNAWFAERVHTIPVCK
EGIRGHTESCSCPLQQSPRADNSAPGTPTRKISASEFDRPLRPIVVKDSEGTVSFLSDSEKKEQMPLTPPRFDHDEGDQCSRLLELVKDISSHLDVT
ALCHKIFLHIHGLISADRYSLFLVCEDSSNDKFLISRLFDVAEGSTLEEVSNNCIRLEWNKGIVGHVAALGEPLNIKDAYEDPRFNAEVDQITGYKT
QSILCMPIKNHREEVVGVAQAINKKSGNGGTATEKDEKDFAAYLAFCGIVLHNAQLYETSLLENKRNQVLLDLASLIFEEQQSLEVILKKIAATIIS
FMQVQKCTIFIVDEDCSDSFSSVFHMECEELEKSSDTLTREHDANKINYMYAQYVKNTMEPLNIPDVSKDKRFPWTTENTGNVNQQCIRSLLCTPIK
NGKKNKVIGVCQLVNKMEENTGKVKPFNRNDEQFLEAFVIFCGLGIQNTQMYEAVERAMAKQMVTLEVLSYHASAAEEETRELQSLAAAVVPSAQTL
KITDFSFSDFELSDLETALCTIRMFTDLNLVQNFQMKHEVLCRWILSVKKNYRKNVAYHNWRHAFNTAQCMFAALKAGKIQNKLTDLEILALLIAAL
SHDLDHRGVNNSYIQRSEHPLAQLYCHSIMEHHHFDQCLMILNSPGNQILSGLSIEEYKTTLKIIKQAILATDLALYIKRRGEFFELIRKNQFNLED
PHQKELFLAMLMTACDLSAITKPWPIQQRIAELVATEFFDQGDRERKELNIEPTDLMNREKKNKIPSMQVGFIDAICLQLYEALTHVSEDCFPLLDG
CRKNRQKWQALAEQQEKMLINGESGQAKRYRGPGIPPARATMTSKVYDPEQRKRMITGPQWWARCKQMNVLDSFINYYDSEKHAENAVIFLHGNAAS
SYLWRHVVPHIEPVARCIIPDLIGMGKSGKSGNGSYRLLDHYKYLTAWFELLNLPKKIIFVGHDWGACLAFHYSYEHQDKIKAIVHAESVVDVIESW
DEWPDIEEDIALIKSEEGEKMVLENNFFVETMLPSKIMRKLEPEEFAAYLEPFKEKGEVRRPTLSWPREIPLVKGGKPDVVQIVRNYNAYLRASDDL
PKMFIESDPGFFSNAIVEGAKKFPNTEFVKVKGLHFSQEDAPDEMGKYIKSFVERVLKNEQ*

Supplementary Text 5: Protein sequence of mNG-GAFa-NLuc constructs.

mNG-GAFa-NLuc

MGSSHHHHHHSSGLVPRGSHMVSKGEEDNMASLPATHELHIFGSINGVDFDMVGQGTGNPNDGYEELNLKSTKGDLQFSPWILVPHIGYGFHQYLPY
PDGMSPFQAAMVDGSGYQVHRTMQFEDGASLTVNYRYTYEGSHIKGEAQVKGTGFPADGPVMTNSLTAADWCRSKKTYPNDKTIISTFKWSYTTGNG
KRYRSTARTTYTFAKPMAANYLKNQPMYVFRKTELKHSKTELNFKEWQKAFTDVMGMDELYKGTGISSHLDVTALCHKIFLHIHGLISADRYSLFLV
CEDSSNDKFLISRLFDVAEGSTLEEVSNNCIRLEWNKGIVGHVAALGEPLNIKDAYEDPRFNAEVDQITGYKTQSILCMPIKNHREEVVGVAQAINK
KSGNGGTFTEKDEKDFAAYLAFCGIVLHNAAAMVFTLEDFVGDWRQTAGYNLDQVLEQGGVSSLFQNLGVSVTPIQRIVLSGENGLKIDIHVIIPYE
GLSGDQMGQIEKIFKVVYPVDDHHFKVILHYGTLVIDGVTPNMIDYFGRPYEGIAVFDGKKITVTGTLWNGNKIIDERLINPDGSLLFRVTINGVTG
WRLCERILA*

mNG-GAFa(L267A)-NLuc

MGSSHHHHHHSSGLVPRGSHMVSKGEEDNMASLPATHELHIFGSINGVDFDMVGQGTGNPNDGYEELNLKSTKGDLQFSPWILVPHIGYGFHQYLPY
PDGMSPFQAAMVDGSGYQVHRTMQFEDGASLTVNYRYTYEGSHIKGEAQVKGTGFPADGPVMTNSLTAADWCRSKKTYPNDKTIISTFKWSYTTGNG
KRYRSTARTTYTFAKPMAANYLKNQPMYVFRKTELKHSKTELNFKEWQKAFTDVMGMDELYKGTGISSHLDVTALCHKIFLHIHGLISADRYSLFLV
CEDSSNDKFLISRLFDVAEGSTLEEVSNNCIRLEWNKGIVGHVAALGEPLNIKDAYEDPRFNAEVDQITGYKTQSIACMPIKNHREEVVGVAQAINK
KSGNGGTFTEKDEKDFAAYLAFCGIVLHNAAAMVFTLEDFVGDWRQTAGYNLDQVLEQGGVSSLFQNLGVSVTPIQRIVLSGENGLKIDIHVIIPYE
GLSGDQMGQIEKIFKVVYPVDDHHFKVILHYGTLVIDGVTPNMIDYFGRPYEGIAVFDGKKITVTGTLWNGNKIIDERLINPDGSLLFRVTINGVTG
WRLCERILA*

mNG-GAFa(F295A)-NLuc

MGSSHHHHHHSSGLVPRGSHMVSKGEEDNMASLPATHELHIFGSINGVDFDMVGQGTGNPNDGYEELNLKSTKGDLQFSPWILVPHIGYGFHQYLPY
PDGMSPFQAAMVDGSGYQVHRTMQFEDGASLTVNYRYTYEGSHIKGEAQVKGTGFPADGPVMTNSLTAADWCRSKKTYPNDKTIISTFKWSYTTGNG
KRYRSTARTTYTFAKPMAANYLKNQPMYVFRKTELKHSKTELNFKEWQKAFTDVMGMDELYKGTGISSHLDVTALCHKIFLHIHGLISADRYSLFLV
CEDSSNDKFLISRLFDVAEGSTLEEVSNNCIRLEWNKGIVGHVAALGEPLNIKDAYEDPRFNAEVDQITGYKTQSILCMPIKNHREEVVGVAQAINK
KSGNGGTATEKDEKDFAAYLAFCGIVLHNAAAMVFTLEDFVGDWRQTAGYNLDQVLEQGGVSSLFQNLGVSVTPIQRIVLSGENGLKIDIHVIIPYE
GLSGDQMGQIEKIFKVVYPVDDHHFKVILHYGTLVIDGVTPNMIDYFGRPYEGIAVFDGKKITVTGTLWNGNKIIDERLINPDGSLLFRVTINGVTG
WRLCERILA*

Supplementary Text 6: Protein sequence of miRFP670nano3 -picALuc(E50A) constructs.

miRFP670nano3-picALuc(E50A)

```
MGSSHHHHHHSSGLVPRGSEFGMANLDKMLNTTVTEVRKFLQADRVCVFKFEEDYSGTVSHEAVDDRWISILKTQVQDRYFMETRGEEYVHGRYQAI
ADIYTANLVECYRDLLIEFQVRAILAVPILQGKKLWGLLVAHQLAGPREWQTWEIDFLKQQAVVMGIAIQQSGSAAATENLYAVLQSGFRGSGSAMK
LPGKKLPLEVLKELEANAQKAGCTRGCLICLSHIKCTAKMKKWLPGRCASWEGDKETGQGGIGEAIVDIPEIPGFKELAPMEQFIAQVDLCADCTTG
CLKGLANVKCSALLKKWLPSRCGTDYKDHDGDYKDHDIDYKDDDDKDI*
```

miRFP670nano3(L100A)-picALuc(E50A)

```
MGSSHHHHHHSSGLVPRGSEFGMANLDKMLNTTVTEVRKFLQADRVCVFKFEEDYSGTVSHEAVDDRWISILKTQVQDRYFMETRGEEYVHGRYQAI
ADIYTANLVECYRDLLIEFQVRAIAAVPILQGKKLWGLLVAHQLAGPREWQTWEIDFLKQQAVVMGIAIQQSGSAAATENLYAVLQSGFRGSGSAMK
LPGKKLPLEVLKELEANAQKAGCTRGCLICLSHIKCTAKMKKWLPGRCASWEGDKETGQGGIGEAIVDIPEIPGFKELAPMEQFIAQVDLCADCTTG
CLKGLANVKCSALLKKWLPSRCGTDYKDHDGDYKDHDIDYKDDDDKDI*
```

miRFP670nano3(W125A)-picALuc(E50A)

```
MGSSHHHHHHSSGLVPRGSEFGMANLDKMLNTTVTEVRKFLQADRVCVFKFEEDYSGTVSHEAVDDRWISILKTQVQDRYFMETRGEEYVHGRYQAI
ADIYTANLVECYRDLLIEFQVRAILAVPILQGKKLWGLLVAHQLAGPREAQTWEIDFLKQQAVVMGIAIQQSGSAAATENLYAVLQSGFRGSGSAMK
LPGKKLPLEVLKELEANAQKAGCTRGCLICLSHIKCTAKMKKWLPGRCASWEGDKETGQGGIGEAIVDIPEIPGFKELAPMEQFIAQVDLCADCTTG
CLKGLANVKCSALLKKWLPSRCGTDYKDHDGDYKDHDIDYKDDDDKDI*
```

Supplementary Tables

Supplementary Table 1. Positional mapping of the coevolving residues on the GAFa domain of PDE5A1.

**Coevolving residues**	**Position**
V230	α3
I266	β5
C268	β5
V281	β6
Q283	β6
I285	β6
S289	β6-α5 loop
L267	β5
F295	β6-α5 loop

Supplementary Table 2. Missense, insertion, and deletion variants of the two coevolving residue positions in the GAF domain-containing human PDE5 reported in the gnomAD database [1]. CADD [2], REVEL [3], and PolyPhen [4] prediction scores were used to distinguish potentially harmful variants from benign ones. B, potentially benign; H, potentially harmful.

PDE isoform (Uniport Entry)	GAF domain (Residue location)	Variant (ID)	Allele count (frequency)	CADD score (outcome)	REVEL score (outcome)	PolyPhen score (outcome)
PDE5A (O76074)	GAFa (L267)	-	-	-	-	-
	GAFa (F295)	p.Phe295Val (4-119567093-A-C)	2 (0.000002401)	27.4 (H)	0.803 (H)	1.0 (H)
	GAFb (L452)	p.Leu452Phe (4-119552592-G-A)	1 (7.154e-7)	29.4 (H)	0.782 (H)	0.986 (H)
		p.Leu452Arg (4-119552591-A-C)	1 (7.143e-7)	29.1 (H)	0.837 (H)	0.997 (H)
	GAFb (F484)	p.Phe484Leu (4-119542581-A-G)	1 (0.000001200)	29.3 (H)	0.803 (H)	0.996 (H)

Supplementary Table 3. List of the holo GAFa domain models of human PDE5 that were generated through homology modeling using the holo GAFa domain of mouse PDE5 as a template (PDB ID: 2K31). The model chosen for MD simulation investigation (highlighted in green) has the lowest molpdf and DOPE scores, and the max GA341 score among the generated models. It also has the highest number of residues in the core region of the Ramachandran plot.

**Model**	**Score assessment**			**Ramachandran plot (%)**				**RMSD**
	**molpdf**	**DOPE**	**GA341**	**core**	**allow**	**generous**	**disallow**	
Holo_GAFa_model.B99990001.pdb	828	-14487	1	92.5	6	1.5	0	0.387
Holo_GAFa_model.B99990002.pdb	715	-14717	1	94	3.8	2.3	0	0.377
Holo_GAFa_model.B99990003.pdb	791	-14524	1	93.2	4.5	1.5	0.8	0.37
Holo_GAFa_model.B99990004.pdb	824	-14478	1	93.2	4.5	1.5	0.8	0.395
Holo_GAFa_model.B99990005.pdb	789	-14399	1	93.2	4.5	1.5	0.8	0.374
Holo_GAFa_model.B99990006.pdb	885	-14360	1	93.2	4.5	2.3	0	0.403
Holo_GAFa_model.B99990007.pdb	825	-14671	1	93.2	4.5	2.3	0	0.391
Holo_GAFa_model.B99990008.pdb	760	-14431	1	92.5	6	1.5	0	0.406
Holo_GAFa_model.B99990009.pdb	760	-14599	1	92.5	4.5	2.3	0.8	0.366
Holo_GAFa_model.B99990010.pdb	752	-14510	1	93.2	4.5	2.3	0	0.381
2k31_NL.pdb (template)				88	9	2.3	0.8	0

Supplementary Table 4: Predicted effects of the mutations on the stability, flexibility, and functionality of the GAFa domain.

	GAFa(L267A)		GAFa(F295A)	
**Tool**	**Value**	**Predicted outcome**	**value**	**Predicted outcome**
ΔΔG DynaMut	-3.732 kcal/mol	Destabilizing	-2.172 kcal/mol	Destabilizing
ΔΔG mCSM	-3.066 kcal/mol	Destabilizing	-2.867 kcal/mol	Destabilizing
ΔΔG SDM	-3.89 kcal/mol	Destabilizing	-3.95 kcal/mol	Destabilizing
ΔΔG DUET	-3.716 kcal/mol	Destabilizing	-3.246 kcal/mol	Destabilizing
ΔΔG DeepDDG	-3.424 kcal/mol	Destabilizing	-3.504 kcal/mol	Destabilizing
ΔΔG iDeepDDG	-3.841 kcal/mol	Destabilizing	-3.452 kcal/mol	Destabilizing
ΔΔG Maestro	-2.982 kcal/mol	Destabilizing	-3.183 kcal/mol	Destabilizing
ΔΔG PoPMuSiC	-2.28 kcal/mol	Destabilizing	-3.69 kcal/mol	Destabilizing
ΔΔG I-Mutant 2.0	-3.94 kcal/mol	Destabilizing	-3.74 kcal/mol	Destabilizing
ΔΔG CUPSAT	-4.85 kcal/mol	Destabilizing	- 3.31 kcal/mol	Destabilizing
ΔΔG Mupro	-1.957 kcal/mol	Destabilizing	-1.183 kcal/mol	Destabilizing
ΔΔG ENCoM	-0.582 kcal/mol	Destabilizing	-1.321 kcal/mol	Destabilizing
$\Delta\Delta S_{vib}$ ENCoM	0.728 $kcal.mol^{-1}.K^{-1}$	Increase flexibility	1.652 $kcal.mol^{-1}.K^{-1}$	Increase flexibility
SNAP2	+59	Functionally deleterious	+82	Functionally deleterious

Supplementary Table 5: Number of contacts formed by the distant coevolving residue positions in the WT and mutant PDE5 GAFa domain.

**Interaction**	**WT (L267)**	**WT (F295)**	**L267A**	**F295A**
Polar contacts	2	0	2	0
Weak polar contacts	2	1	3	0
Hydrogen bonds	2	0	2	0
Weak hydrogen bonds	2	1	1	0
Carbonyl interactions	1	0	1	0
Hydrophobic contacts	24	25	5	2

449 # Supplementary Figures

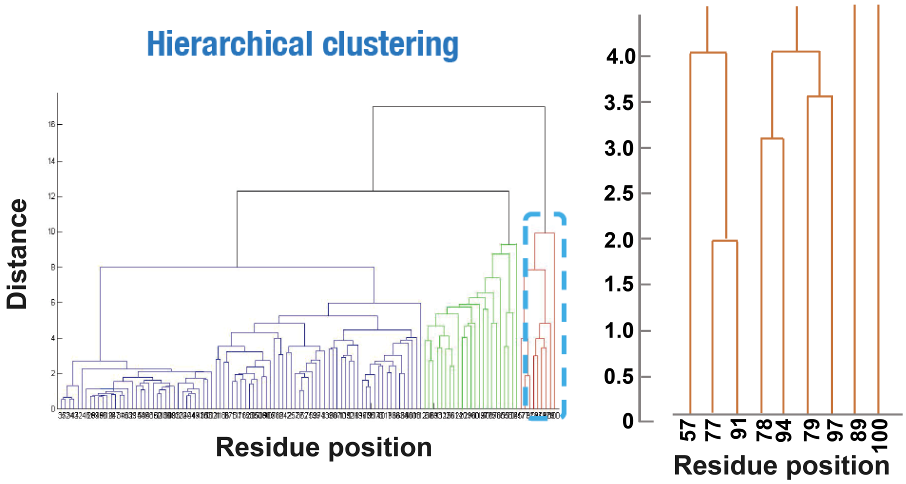

450

451 **Supplementary Figure 1. Hierarchical clustering of the coevolving residue positions in the GAF**
452 **domains based on their pairwise SCA scores.** The cluster of residues with the highest SCA scores is
453 shown in red and zoomed in (right panel).

454

455

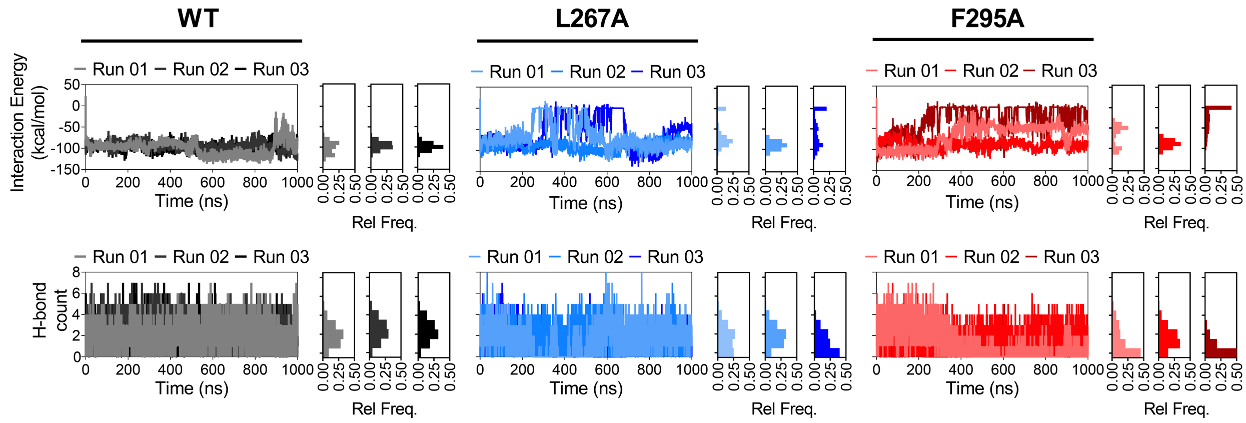

Supplementary Figure 2. L267A and F295A mutations weaken GAFa domain-cGMP interaction. Graphs showing changes in the interaction energy (vdW and electrostatics; upper panel) and number of H-bonds formed between cGMP and GAFa domain (lower panel) obtained from three independent, all-atom, 1000 ns-long MD simulation runs. Outsets represent the frequency distribution of interaction energy (upper panel) and H-bonds (bottom panel) obtained from three independent, all-atom, 1000 ns-long MD simulation runs.

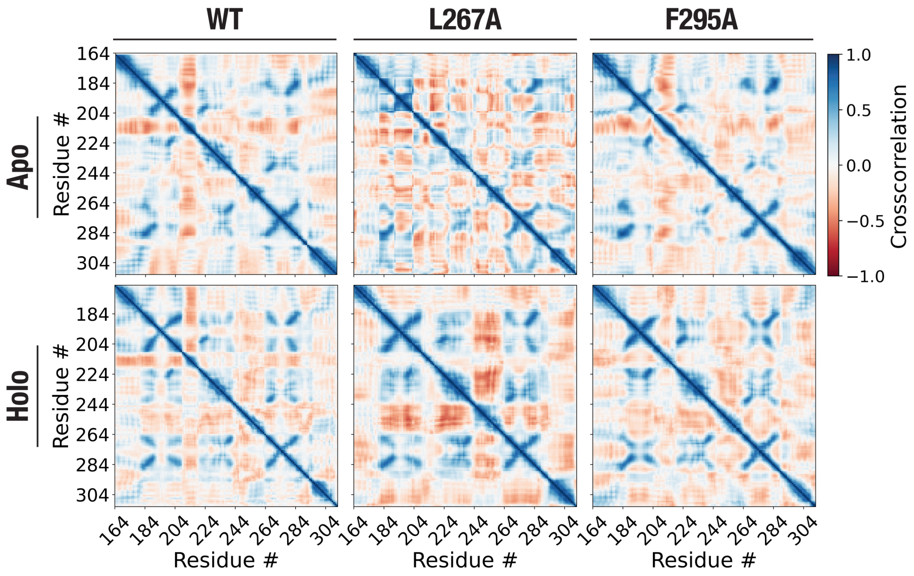

Supplementary Figure 3. Dynamic cross-correlation (DCC) analysis of apo and holo GAFa domains of human PDE5. Heatmap showing average DCC values of the apo (upper panel) and holo (lower panel) GAFa domains (WT and mutants) obtained from three independent, all-atom, 1000 ns-long MD simulation runs.

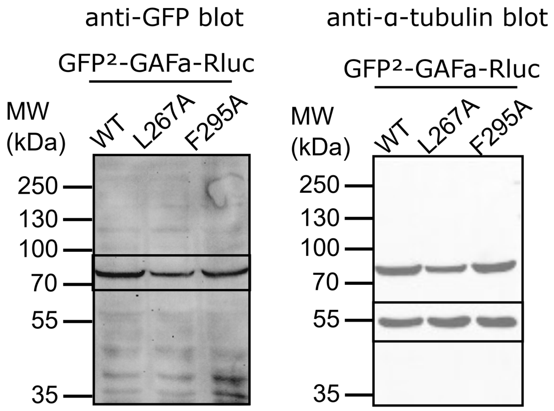

Supplementary Figure 4. Image of the whole blot used for generating Fig. 4D in the main text. The predicted molecular weight of the GAFa domain biosensor is ~87 kDa. WT, wild type. Note the boxes in the blot showing the region used to generate the main text figure.

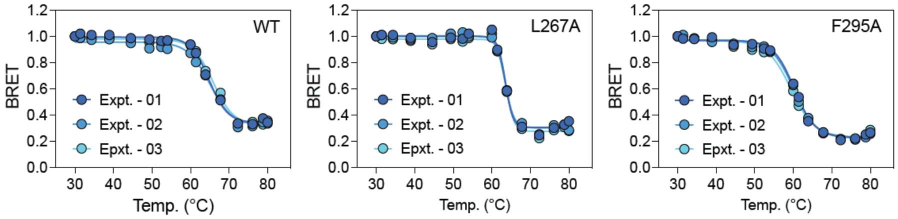

Supplementary Figure 5. Thermal stability of the GAFa domain. Graph showing the effect of temperature on the stability of the WT and mutant GAFa domains inferred from measuring the BRET ratio between mNG and NLuc in the WT (left) and mutant (middle and right) mNG-GAFa-NLuc biosensors. Data shown are from three independent experiments and fitted to a Boltzmann sigmoidal model to determine melting temperatures (T_m) for each protein that were reported in Fig. 4I.

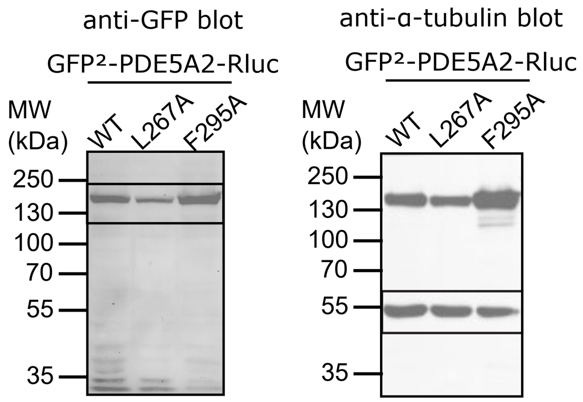

Supplementary Figure 6. Image of the whole blot used for generating Fig. 5B in the main text. The predicted molecular weight of the full-length PDE5A2 biosensor is ~162 kDa. WT, wild type. Note the boxes in the blot showing the region used to generate the main text figure.

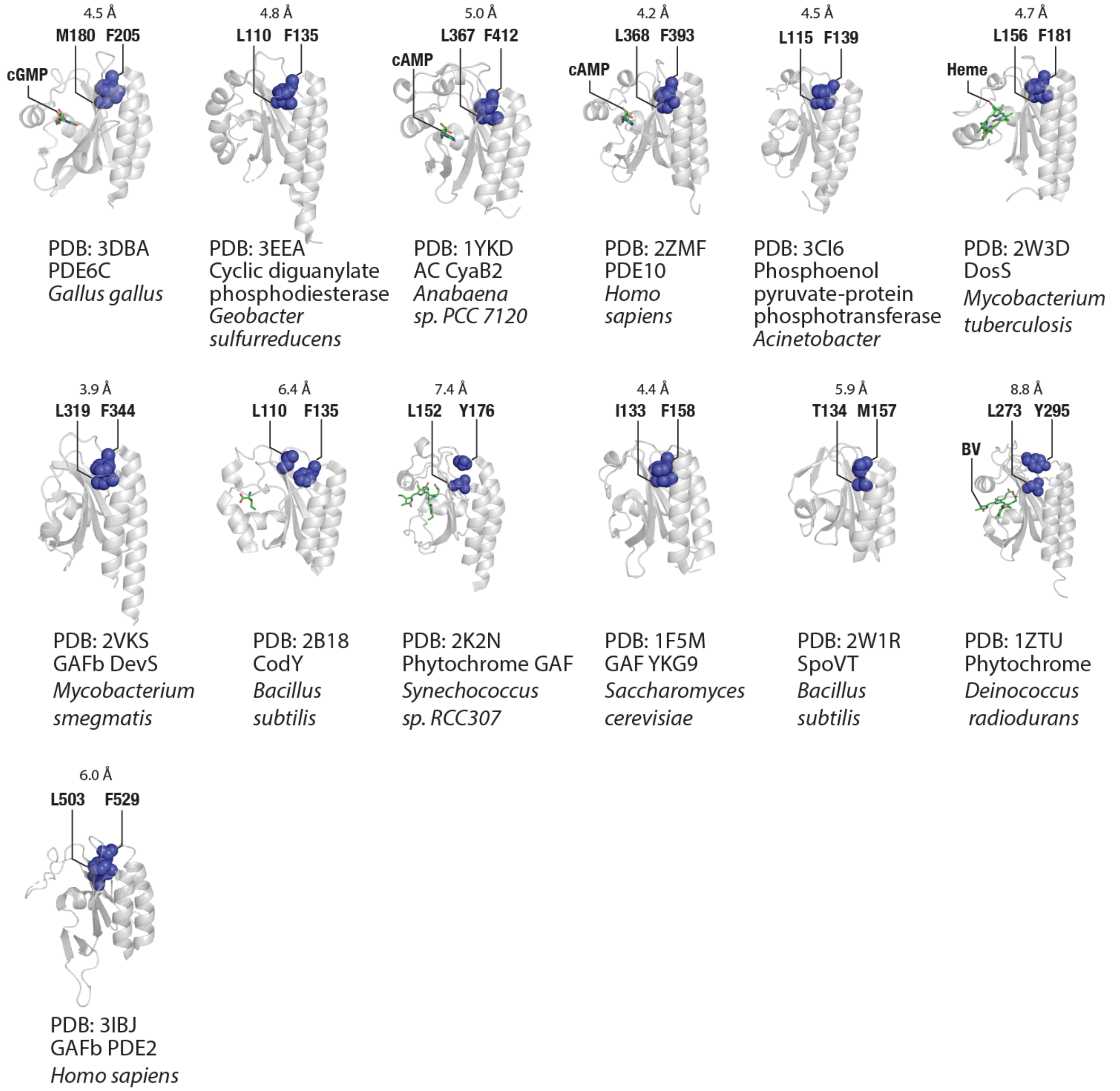

Supplementary Figure 7. Cartoon representations of GAF domain structures obtained from the RCSB database including their PDB code, the protein name, and the species name. The ligand, if present in the structure, is represented as sticks, and the distant coevolving residue positions (equivalent to L267 and F295 in the PDE5 GAFa domain) are represented as dark blue spheres. Note the conservation and proximity of the two distant coevolving residue positions in the structures irrespective of the presence of the ligand in the structures.

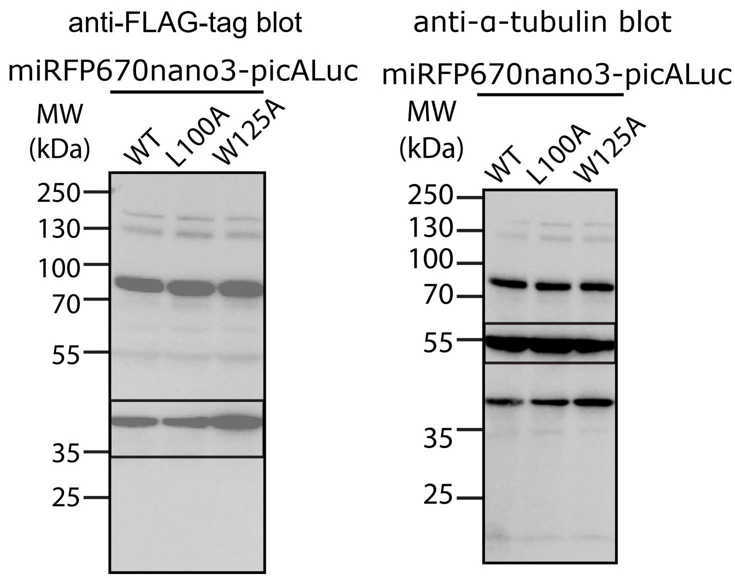

Supplementary Figure 8. Image of the whole blot used for generating Fig. 5l in the main text. The predicted molecular weight of the miRFP3670nano3 construct is ~38 kDa. WT, wild type. Note the boxes in the blot showing the region used to generate the main text figure.

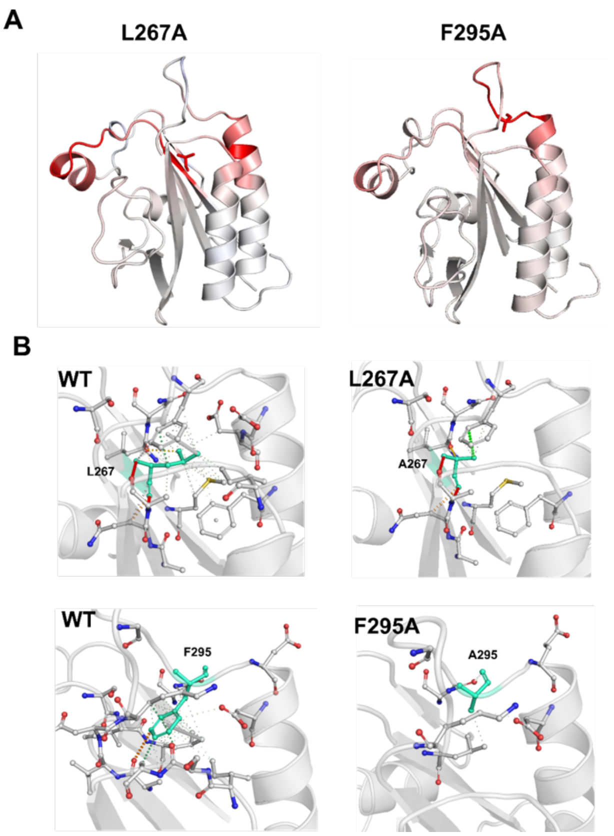

Supplementary Figure 9. Mutating the coevolving residues disrupts their inter-residue interactions. (A) Change in the vibrational entropy energy (ΔΔSvib) between WT and mutant GAFa domains. Red and blue colors represent gains in flexibility and rigidity, respectively. Mutated residues are shown in red sticks. (B) Inter-residue interactions formed by the mutated positions in the WT (left) and mutant (right) GAFa domains. WT and mutant residues are colored in cyan and represented as sticks alongside the surrounding interacting residues.

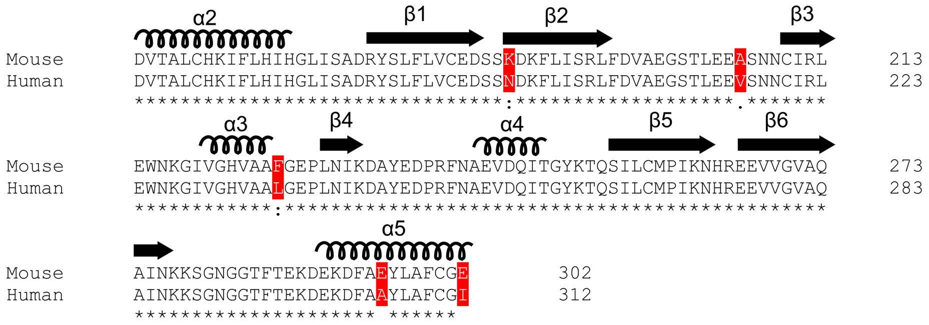

Supplementary Figure 10. Amino acid sequence alignment of mouse and human PDE5 GAFa domains. Differences in the amino acid sequences are highlighted in red. Secondary structure elements (α helices and β sheets) are indicated above the sequences.

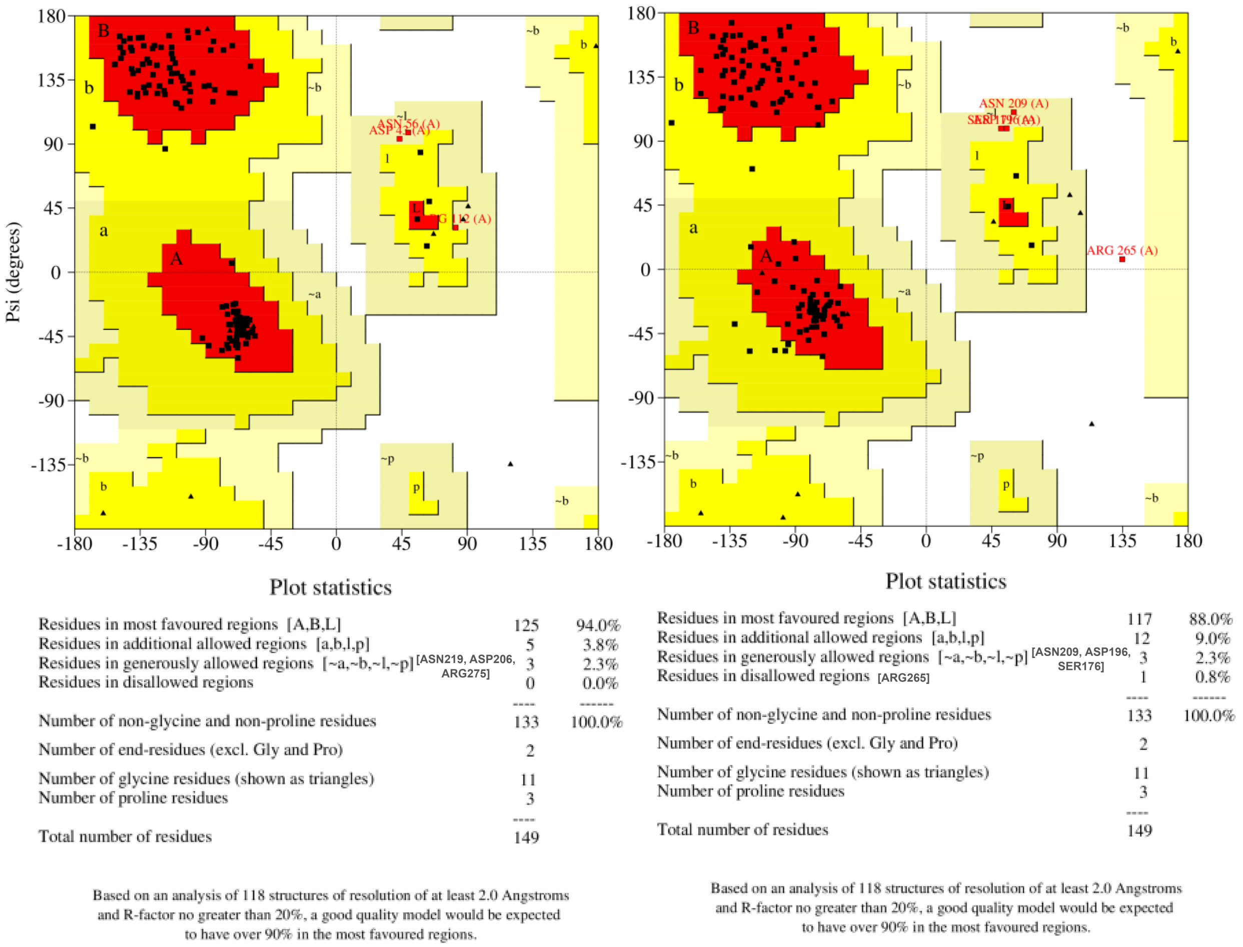

Supplementary Figure 11. Ramachandran plot of the holo GAFa domain of human PDE5. The structural model of the human GAFa domain of PDE5 (left panel) spanning residues from D164 to I312 was generated from the mouse holo PDE5-GAFa model (PDB: 2K31) (right panel). The core regions (red) represent the most favorable combination of phi-psi values. The percentage of residues in the core is 94%, depicting a better stereochemical quality compared to the mouse template structure, which has 88% of residues in the core region.

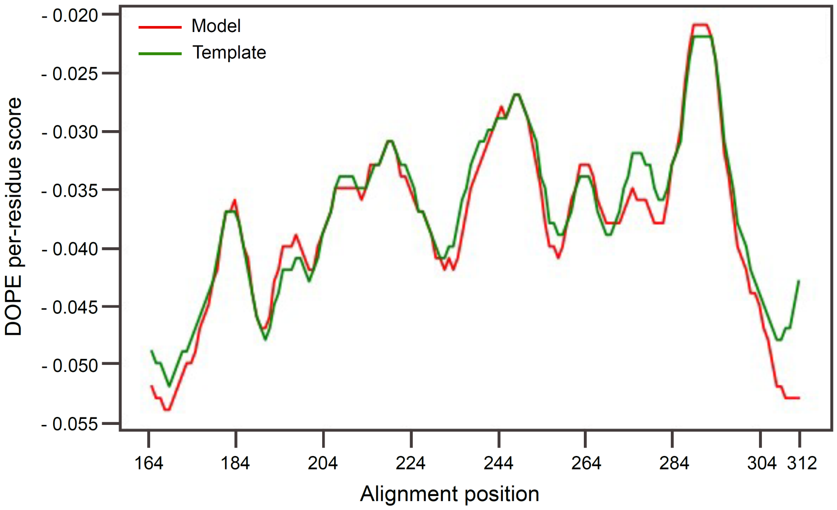

Supplementary Figure 12. Quality Estimation of the modeled holo GAFa domain of human PDE5 (Red) compared to the modeling template GAFa domain of mouse PDE5 (Green) using discrete optimized potential energy (DOPE) score profiling.

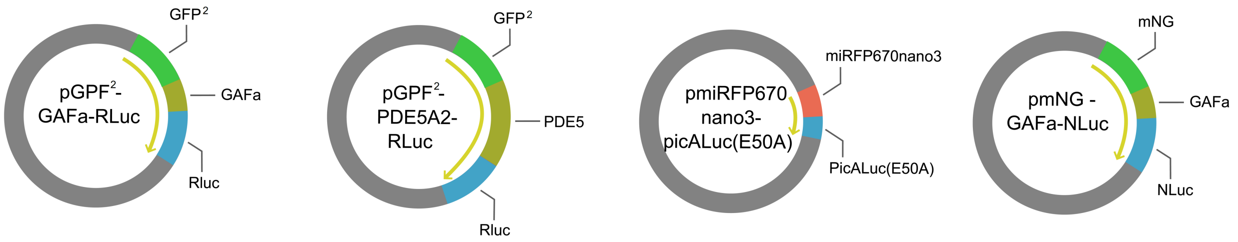

Supplementary Figure 13. Schematic depicting the plasmids used for generating the GAFa (left), full-length PDE5A2 (second left), miRFP670nano3 (second right), and GAFa thermal stability (right) WT and mutant protein constructs.

References

1. Karczewski, K.J., et al., *The mutational constraint spectrum quantified from variation in 141,456 humans.* Nature, 2020. **581**(7809): p. 434-443.
2. Rentzsch, P., et al., *CADD: predicting the deleteriousness of variants throughout the human genome.* Nucleic acids research, 2019. **47**(D1): p. D886-D894.
3. Ioannidis, N.M., et al., *REVEL: An Ensemble Method for Predicting the Pathogenicity of Rare Missense Variants.* American journal of human genetics, 2016. **99**(4): p. 877-885.
4. Adzhubei, I.A., et al., *A method and server for predicting damaging missense mutations.* Nature methods, 2010. **7**(4): p. 248-249.
5. Niroula, A. and M. Vihinen, *How good are pathogenicity predictors in detecting benign variants?* PLoS computational biology, 2019. **15**(2): p. e1006481.
6. Martin, F.J., et al., *Ensembl 2023.* Nucleic acids research, 2023. **51**(D1): p. D933-D941.
7. Rodrigues, C.H., D.E. Pires, and D.B. Ascher, *DynaMut: predicting the impact of mutations on protein conformation, flexibility and stability.* Nucleic acids research, 2018. **46**(W1): p. W350-W355.
8. Pires, D.E., D.B. Ascher, and T.L. Blundell, *mCSM: predicting the effects of mutations in proteins using graph-based signatures.* Bioinformatics (Oxford, England), 2014. **30**(3): p. 335-342.
9. Worth, C.L., R. Preissner, and T.L. Blundell, *SDM--a server for predicting effects of mutations on protein stability and malfunction.* Nucleic acids research, 2011. **39**(Web Server issue): p. W215-W222.
10. Pires, D.E., D.B. Ascher, and T.L. Blundell, *DUET: a server for predicting effects of mutations on protein stability using an integrated computational approach.* Nucleic acids research, 2014. **42**(Web Server issue): p. W314-W319.
11. Cao, H., et al., *DeepDDG: Predicting the Stability Change of Protein Point Mutations Using Neural Networks.* Journal of chemical information and modeling, 2019. **59**(4): p. 1508-1514.
12. Laimer, J., et al., *MAESTROweb: a web server for structure-based protein stability prediction.* Bioinformatics (Oxford, England), 2016. **32**(9): p. 1414-1416.
13. Gilis, D. and M. Rooman, *PoPMuSiC, an algorithm for predicting protein mutant stability changes: application to prion proteins.* Protein engineering, 2000. **13**(12): p. 849-856.
14. Capriotti, E., P. Fariselli, and R. Casadio, *I-Mutant2. 0: predicting stability changes upon mutation from the protein sequence or structure.* Nucleic acids research, 2005. **33**(Web Server issue): p. W306-W310.
15. Parthiban, V., M.M. Gromiha, and D. Schomburg, *CUPSAT: prediction of protein stability upon point mutations.* Nucleic acids research, 2006. **34**(Web Server issue): p. W239-W242.
16. Cheng, J., A. Randall, and P. Baldi, *Prediction of protein stability changes for single-site mutations using support vector machines.* Proteins, 2006. **62**(4): p. 1125-1132.
17. Frappier, V., M. Chartier, and R.J. Najmanovich, *ENCoM server: exploring protein conformational space and the effect of mutations on protein function and stability.* Nucleic acids research, 2015. **43**(W1): p. W395-W400.
18. Jubb, H.C., et al., *Arpeggio: A Web Server for Calculating and Visualising Interatomic Interactions in Protein Structures.* Journal of molecular biology, 2017. **429**(3): p. 365-371.
19. Hecht, M., Y. Bromberg, and B. Rost, *Better prediction of functional effects for sequence variants.* BMC genomics, 2015. **16**(Suppl 8): p. S1.
20. Brandes, N., et al., *Genome-wide prediction of disease variant effects with a deep protein language model.* Nat Genet, 2023. **55**(9): p. 1512-1522.
