# Supplementary material for: Coevolving residues distant from the ligand binding site are involved in GAF domain function
Source: Commun Chem. 2025 Apr 7;8:107. doi: 10.1038/s42004-025-01447-9 (PMC11977230; doi:10.1038/s42004-025-01447-9)
Supplement: Supplementary file 3 — Description of Additional Supplementary Files [file 42004_2025_1447_MOESM3_ESM.pdf]

Description of Additional Supplementary Files

File name: Supplementary Movie 1

Description: Positional mapping of coevolving cluster of residues on the holo GAFa domain of PDE5 (PDB: 2K31).

File name: Supplementary Movie 2

Description: Dynamics of L267 and F295 obtained from the NMR models of PDE5 holo GAFa domain (PDB: 2K31).

File name: Supplementary Movie 3

Description: Comparison of L267 and F295 dynamics in the holo (blue spheres) and apo (purple spheres) GAFa domain of PDE5.

File name: Supplementary Movie 4

Description: MD simulation - Apo WT GAFa domain Run 01

File name: Supplementary Movie 5

Description: MD simulation – Apo WT GAFa domain Run 02

File name: Supplementary Movie 6

Description: MD simulation – Apo WT GAFa domain Run 03

File name: Supplementary Movie 7

Description: MD simulation – Apo L267A GAFa domain Run 01

File name: Supplementary Movie 8

Description: MD simulation – Apo L267A GAFa domain Run 02

File name: Supplementary Movie 9

Description: MD simulation – Apo L267A GAFa domain Run 03

File name: Supplementary Movie 10

Description: MD simulation – Apo F295A GAFa domain Run 01

File name: Supplementary Movie 11

Description: MD simulation – Apo F295A GAFa domain Run 02

File name: Supplementary Movie 12

Description: MD simulation – Apo F295A GAFa domain Run 03

File name: Supplementary Movie 13

Description: MD simulation - Holo WT GAFa domain Run 01

File name: Supplementary Movie 14

Description: MD simulation – Holo WT GAFa domain Run 02

File name: Supplementary Movie 15

Description: MD simulation – Holo WT GAFa domain Run 03

File name: Supplementary Movie 16

Description: MD simulation – Holo L267A GAFa domain Run 01

File name: Supplementary Movie 17

Description: MD simulation – Holo L267A GAFa domain Run 02

File name: Supplementary Movie 18

Description: MD simulation – Holo L267A GAFa domain Run 03

File name: Supplementary Movie 19

Description: MD simulation – Holo F295A GAFa domain Run 01

File name: Supplementary Movie 20

Description: MD simulation – Holo F295A GAFa domain Run 02

File name: Supplementary Movie 21

Description: MD simulation – Holo F295A GAFa domain Run 03

File name: Supplementary Data 1

Description: Apo_WT-pdb

File name: Supplementary Data 2

Description: Holo_WT-pdb

File name: Supplementary Data 3

Description: Apo_L267A-pdb

File name: Supplementary Data 4

Description: Holo_L267A-pdb

File name: Supplementary Data 5

Description: Apo_F295A-pdb

File name: Supplementary Data 6

Description: Holo_F295A-pdb
