# Supplementary material for: Coevolving residues distant from the ligand binding site are involved in GAF domain function
Source: Commun Chem. 2025 Apr 7;8:107. doi: 10.1038/s42004-025-01447-9 (PMC11977230; doi:10.1038/s42004-025-01447-9)
Supplement: Supplementary file 6 — nr-reporting-summary [file 42004_2025_1447_MOESM6_ESM.pdf]

Corresponding author(s): Kabir H Biswas

Last updated by author(s): Feb 4, 2025

Reporting Summary

Nature Portfolio wishes to improve the reproducibility of the work that we publish. This form provides structure for consistency and transparency in reporting. For further information on Nature Portfolio policies, see our Editorial Policies and the Editorial Policy Checklist.

Statistics

For all statistical analyses, confirm that the following items are present in the figure legend, table legend, main text, or Methods section.

n/a	Confirmed	
☐	☒	The exact sample size (n) for each experimental group/condition, given as a discrete number and unit of measurement
☐	☒	A statement on whether measurements were taken from distinct samples or whether the same sample was measured repeatedly
☐	☒	The statistical test(s) used AND whether they are one- or two-sided<br>*Only common tests should be described solely by name; describe more complex techniques in the Methods section.*
☒	☐	A description of all covariates tested
☒	☐	A description of any assumptions or corrections, such as tests of normality and adjustment for multiple comparisons
☒	☐	A full description of the statistical parameters including central tendency (e.g. means) or other basic estimates (e.g. regression coefficient) AND variation (e.g. standard deviation) or associated estimates of uncertainty (e.g. confidence intervals)
☒	☐	For null hypothesis testing, the test statistic (e.g. F, t, r) with confidence intervals, effect sizes, degrees of freedom and P value noted<br>*Give P values as exact values whenever suitable.*
☒	☐	For Bayesian analysis, information on the choice of priors and Markov chain Monte Carlo settings
☒	☐	For hierarchical and complex designs, identification of the appropriate level for tests and full reporting of outcomes
☒	☐	Estimates of effect sizes (e.g. Cohen's d, Pearson's r), indicating how they were calculated

Our web collection on statistics for biologists contains articles on many of the points above.

Software and code

Policy information about availability of computer code

Data collection	1. NAMD software v2.13<br>2. MODELLER (10.1 release, Mar. 18, 2021)<br>3. CHARMM-GUI webserver<br>4. PyMOL
Data analysis	1. VMD software v1.9.3 and v1.9.4<br>2. CaFE 1.0 plugin in VMD v1.9.4<br>3. PyMOL

For manuscripts utilizing custom algorithms or software that are central to the research but not yet described in published literature, software must be made available to editors and reviewers. We strongly encourage code deposition in a community repository (e.g. GitHub). See the Nature Portfolio guidelines for submitting code & software for further information.

Data

Policy information about availability of data

All manuscripts must include a data availability statement. This statement should provide the following information, where applicable:

- Accession codes, unique identifiers, or web links for publicly available datasets
- A description of any restrictions on data availability
- For clinical datasets or third party data, please ensure that the statement adheres to our policy

All the relevant data of this study are available within the manuscript and its Supplementary Information file.

Research involving human participants, their data, or biological material

Policy information about studies with human participants or human data. See also policy information about sex, gender (identity/presentation), and sexual orientation and race, ethnicity and racism.

Reporting on sex and gender	N/A
Reporting on race, ethnicity, or other socially relevant groupings	N/A
Population characteristics	N/A
Recruitment	N/A
Ethics oversight	N/A

Note that full information on the approval of the study protocol must also be provided in the manuscript.

Field-specific reporting

Please select the one below that is the best fit for your research. If you are not sure, read the appropriate sections before making your selection.

☒ Life sciences ☐ Behavioural & social sciences ☐ Ecological, evolutionary & environmental sciences

For a reference copy of the document with all sections, see nature.com/documents/nr-reporting-summary-flat.pdf

Life sciences study design

All studies must disclose on these points even when the disclosure is negative.

Sample size	MD simulations were performed in triplicates and in vitro assays were performed either three times or four times and have been reported in the manuscript.
Data exclusions	N/A
Replication	Experiments reported in the manuscript have been repeated either three times or four times
Randomization	N/A
Blinding	N/A

Reporting for specific materials, systems and methods

We require information from authors about some types of materials, experimental systems and methods used in many studies. Here, indicate whether each material, system or method listed is relevant to your study. If you are not sure if a list item applies to your research, read the appropriate section before selecting a response.

Materials & experimental systems

n/a	Involved in the study	
☐	☒	Antibodies
☐	☒	Eukaryotic cell lines
☒	☐	Palaeontology and archaeology
☒	☐	Animals and other organisms
☒	☐	Clinical data
☒	☐	Dual use research of concern
☒	☐	Plants

Methods

n/a	Involved in the study	
☒	☐	ChIP-seq
☒	☐	Flow cytometry
☒	☐	MRI-based neuroimaging

Antibodies

Antibodies used	We utilized the following commercially available antibodies in the current study:<br>1. Anti-GFP antibody (mouse IgG2a κ mAb, Santa Cruz Biotechnology, USA – SC-9996; 1:500 dilution)<br>2. Anti-FLAG-tag antibody (mouse IgG2a mAB, Sino Biological, China – 109143-MM13; 1:10000 dilution)<br>3. Anti-α-tubulin antibody (mouse IgG2b mAB, Proteintech, USA – 66031-1; 1:200000)<br>3. Secondary antibody (anti-Mouse IgG:HRP Donkey pAb; ECM Biosciences, USA – MS3001; 1:20000 dilution)
Validation	Validation/details for the respective antibodies are available in the indicated weblinks:<br>1. Anti-GFP antibody - https://www.scbt.com/p/gfp-antibody-b-2<br>2. AAnti-FLAG-tag antibody - https://www.sinobiological.com/antibodies/flag-109143-mm13<br>3. Anti-α-tubulin antibody - https://www.ptglab.com/products/tubulin-Alpha-Antibody-66031-1-Ig.htm<br>4. Secondary antibody - https://ecmbio.com/products/ms3001

Eukaryotic cell lines

Policy information about cell lines and Sex and Gender in Research

Cell line source(s)	HEK293T
Authentication	HEK293T is a commonly used cell line and we have not authenticated the cell line other than the visual inspection of the cell morphology.
Mycoplasma contamination	Not tested
Commonly misidentified lines<br>(See ICLAC register)	N/A

Plants

Seed stocks	N/A
Novel plant genotypes	N/A
Authentication	N/A
